# Supplementary material for: Tropical wetlands and land use changes: The case of oil palm in neotropical riverine floodplains
Source: PLoS One. 2022 May 12;17(5):e0266677. doi: 10.1371/journal.pone.0266677 (PMC9098095; doi:10.1371/journal.pone.0266677)
Supplement: S1 Table — (DOCX) [file pone.0266677.s001.docx]

**S1 Table A. Metrics used at class and landscape level to quantify fragmentation [67,68,69]**

| Metrics | Level of analysis | Description |
| --- | --- | --- |
| **Fragmentation** |  |  |
| Number of Patches (NP) | Class/landscape | NP equals the number of patches of the class |
| Patches density (PD) | Class/landscape | PD equals the number of patches of the corresponding patch type (NP) divided by total landscape area, multiplied by 10,000 and 100 (to convert to 100 ha) |
| Total Edge (TE) | Class/landscape | TE equals the sum of the lengths (m) of all edge segments in the class |
| Edge Density (DE) | Class/landscape | Total length of edge involving the corresponding land use/land cover class divided by total area (ha) |
| Largest Patch Index (LPI) | Class/landscape | Equals the percentage of the landscape comprised by the largest patch |
| Percentage of Landscape (PLAND) | Class | Percentage the landscape comprised of the corresponding patch type |
| Landscape shape index (LSI) | Class/landscape | This index measures the perimeter-to-area ratio for the landscape as a whole. Average complexity of the landscape as a whole |
| Interspersion-Juxtaposition Index (IJI) | Class/landscape | A measure of adjacency of patches determined by dividing the length between patch edges by the number of patches within a landscape in percentage. Lower values (approaching 0%) indicate that a patch is adjacent to only one other patch and higher values indicate that a patch is in similar proximity to multiple patches within a landscape |
| Effective Mesh Size (MESH). | Landscape | Expresses the size of the areas when divided into S areas (each of the same size At/S) with the same degree of landscape division (in meters) |
| **Diversity and heterogeneity** |  |  |
| Contagion index (CONTAG) | Landscape | Are measures of the placement of patch types relative to other patch types  Large CONTAG reflects clumping patches. Small CONTAG reflects a landscape dissected |
| Simpson index (SIDI) | Landscape | Combines the richness and evenness of habitat classes. Its value represents the probability that any two random points in the landscape correspond to different class types. Values approaching zero represent lower diversity. Values close to one represent different patches and the proportional distribution becomes more equitable. |
| Shannon index (SHDI) | Landscape | Expresses heterogeneity when SHEI values are higher. The SHDI value increases with the number of classes and the uniformity of their proportions. In two landscapes with the same number of classes, the higher SHDI will apply to the landscape in which the proportions of classes are more similar |

**S1 Table B. Class-level landscape metrics for the ten subsections (W1-W10) during 2001-2017**

| Window | Year | Cover type | Code | CA | PLAND | NP | PD | LPI | TE | ED | MESH | IJI | LSI |
| --- | --- | --- | --- | --- | --- | --- | --- | --- | --- | --- | --- | --- | --- |
| W1 | 2001 | Rain Forest | RF | 28937.07 | 50.23 | **988.00** | 1.71 | 37.09 | 3036600 | 52.71 | **7992.08** | 75.88 | 45.34 |
| W1 | 2001 | Mangrove | MG | 5335.74 | 9.26 | 1372.00 | 2.38 | 1.39 | 1216440 | 21.11 | 18.19 | 21.19 | 41.92 |
| W1 | 2001 | Hidrophytic vegetation | HV | 574.38 | 0.99 | 510.00 | 0.88 | 0.06 | 226170 | 3.92 | 0.09 | 65.89 | 23.61 |
| W1 | 2001 | Shrubland | SHR | 6568.92 | 83.94 | 1748.00 | 3.03 | 1.45 | 1391190 | 24.15 | 18.87 | 61.63 | 43.33 |
| W1 | 2001 | Secondary vegetation | SV | 8051.22 | 13.97 | 2264.00 | 3.93 | 0.76 | 2038440 | 35.38 | 14.15 | 72.07 | 56.95 |
| W1 | 2001 | Bare Soil | BS | 952.92 | 1.65 | 333.00 | 0.57 | 0.20 | 226830 | 3.93 | 0.72 | 51.26 | 18.41 |
| W1 | 2001 | Agricultural | AGT | 7179.75 | 12.46 | 1471.00 | 2.55 | 0.97 | 1494630 | 25.94 | 70.27 | 70.27 | 44.41 |
| W1 | 2017 | Rain forest | RF | 18408.24 | 31.96 | **1056.00** | 1.83 | 4.04 | 2313000 | 40.16 | **262.29** | 65.09 | 43.38 |
| W1 | 2017 | Mangrove | MG | 3734.10 | 6.48 | 1026.00 | 1.78 | 0.73 | 953880 | 16.56 | 7.56 | 57.24 | 39.24 |
| W1 | 2017 | Shrubland | SHR | 9112.23 | 15.82 | 1540.00 | 2.67 | 1.95 | 1729350 | 30.02 | 49.80 | 64.90 | 45.66 |
| W1 | 2017 | Hidrophytic vegetation | HV | 277.65 | 0.48 | 431.00 | 0.75 | 0.02 | 147360 | 2.56 | 0.02 | 59.44 | 22.07 |
| W1 | 2017 | Aquatic ecosystem | AQ | 51.03 | 0.09 | 18.00 | 0.03 | 0.02 | 14220 | 0.25 | 0.01 | 40.35 | 4.94 |
| W1 | 2017 | Secondary vegetation | SV | 18767.16 | 32.58 | 1241.00 | 2.15 | 12.43 | 2714970 | 47.13 | 961.96 | 75.90 | 49.92 |
| W1 | 2017 | Agricultural | AGT | 4862.34 | 8.44 | 902.00 | 1.57 | 1.15 | 969450 | 16.83 | 14.47 | 53.39 | 34.97 |
| W1 | 2017 | Bare soil | BS | 507.51 | 0.88 | 151.00 | 0.26 | 0.11 | 112500 | 1.95 | 0.20 | 68.23 | 12.48 |
| **W1** | **2017** | **Oil palm crop** | **OPC** | **1879.74** | **3.26** | **210.00** | **0.36** | **0.45** | **278670** | **4.84** | **1.82** | **61.41** | **16.06** |
| W2 | 2001 | Rain forest | RF | **15428.79** | 26.79 | **1122.00** | 1.95 | 9.87 | 1746480 | 30.32 | **617.40** | 53.92 | 35.50 |
| W2 | 2001 | Dry forest | DF | 22.68 | 0.04 | 32.00 | 0.06 | 0.01 | 13200 | 0.23 | 0.00 | 54.61 | 6.88 |
| W2 | 2001 | Shrubland | SHR | 3848.22 | 6.68 | 1150.00 | 2.00 | 0.74 | 882300 | 15.32 | 8.69 | 80.53 | 35.84 |
| W2 | 2001 | Hidrophytic vegetation | HV | 4432.23 | 7.69 | 1248.00 | 2.17 | 0.61 | 1172550 | 20.36 | 5.99 | 46.12 | 44.24 |
| W2 | 2001 | Aquatic ecosystem | AQ | 1460.79 | 2.54 | 98.00 | 0.17 | 0.76 | 218310 | 3.79 | 6.26 | 67.69 | 14.61 |
| W2 | 2001 | Secondary vegetation | SV | 25340.13 | 43.99 | 940.00 | 1.63 | 18.31 | 2886840 | 50.12 | 2414.47 | 77.42 | 46.01 |
| W2 | 2001 | Agricultural | AGT | 4293.99 | 7.45 | 849.00 | 1.47 | 0.96 | 931080 | 16.16 | 14.65 | 58.91 | 35.89 |
| W2 | 2001 | Bare soil | BS | 2773.17 | 4.81 | 331.00 | 0.57 | 0.60 | 409500 | 7.11 | 6.34 | 61.11 | 19.51 |
| W2 | 2017 | Rain forest | RF | **11752.74** | 20.40 | **705.00** | 1.22 | 8.69 | 1083360 | 18.81 | **480.59** | 61.68 | 25.32 |
| W2 | 2017 | Shrubland | SHR | 8374.68 | 14.54 | 1173.00 | 2.04 | 3.80 | 1398240 | 24.28 | 96.16 | 76.10 | 38.52 |
| W2 | 2017 | Hidrophytic vegetation | HV | 6636.87 | 11.52 | 1562.00 | 2.71 | 1.00 | 1467480 | 25.48 | 14.34 | 60.38 | 45.35 |
| W2 | 2017 | Aquatic ecosystem | AQ | 1229.31 | 2.13 | 65.00 | 0.11 | 0.76 | 197820 | 3.43 | 5.04 | 53.64 | 14.41 |
| W2 | 2017 | Secondary vegetation | SV | 22083.12 | 38.34 | 820.00 | 1.42 | 14.76 | 2167860 | 37.64 | 1499.75 | 76.00 | 37.12 |
| W2 | 2017 | Agricultural | AGT | 3753.63 | 6.52 | 437.00 | 0.76 | 0.76 | 577560 | 10.03 | 10.74 | 51.80 | 23.95 |
| W2 | 2017 | Bare soil | BS | 128.97 | 0.22 | 50.00 | 0.09 | 0.03 | 31800 | 0.55 | 0.02 | 67.18 | 7.20 |
| **W2** | **2017** | **Oil palm crop** | **OPC** | **3640.68** | **6.32** | **6.00** | **0.01** | **5.92** | **70500** | **1.22** | **202.43** | **65.52** | **2.92** |
| W3 | 2001 | Rain forest | RF | **4041.00** | 7.02 | **1484.00** | 2.58 | 0.50 | 1144020 | 19.86 | **5.48** | 52.23 | 45.43 |
| W3 | 2001 | Dry forest | DF | 265.14 | 0.46 | 366.00 | 0.64 | 0.04 | 136560 | 2.37 | 0.02 | 53.93 | 20.92 |
| W3 | 2001 | Shrubland | SHR | 1791.09 | 3.11 | 1985.00 | 3.45 | 0.11 | 849690 | 14.75 | 0.28 | 63.67 | 50.20 |
| W3 | 2001 | Hidrophytic vegetation | HV | 1309.23 | 2.27 | 770.00 | 1.34 | 0.10 | 483720 | 8.40 | 0.41 | 40.53 | 33.52 |
| W3 | 2001 | Aquatic ecosystem | AQ | 4515.93 | 7.84 | 288.00 | 0.50 | 3.77 | 400230 | 6.95 | 89.18 | 69.63 | 15.28 |
| W3 | 2001 | Secondary vegetation | SV | 43019.82 | 74.69 | 313.00 | 0.54 | 72.49 | 2603640 | 45.20 | 30267.75 | 85.90 | 32.14 |
| W3 | 2001 | Agricultural | AGT | 2206.80 | 3.83 | 1261.00 | 2.19 | 0.28 | 772050 | 13.40 | 1.00 | 39.42 | 41.15 |
| W3 | 2001 | Bare Soil | BS | 450.99 | 0.78 | 341.00 | 0.59 | 0.03 | 175410 | 3.05 | 0.05 | 52.86 | 20.62 |
| W3 | 2017 | Rain forest | RF | **5953.95** | 10.34 | **1508.00** | 2.62 | 0.84 | 1349940 | 23.44 | **11.34** | 59.74 | 44.06 |
| W3 | 2017 | Shrubland | SHR | 2408.31 | 4.18 | 1630.00 | 2.83 | 0.47 | 907680 | 15.76 | 1.82 | 80.53 | 46.39 |
| W3 | 2017 | Hidrophytic vegetation | HV | 8466.03 | 14.70 | 3367.00 | 5.85 | 0.73 | 2608470 | 45.29 | 7.93 | 68.09 | 71.32 |
| W3 | 2017 | Aquatic ecosystem | AQ | 4487.85 | 7.79 | 164.00 | 0.28 | 3.83 | 332850 | 5.78 | 91.87 | 71.76 | 12.83 |
| W3 | 2017 | Secondary vegetation | SV | 29790.27 | 51.72 | 865.00 | 1.50 | 41.82 | 3165510 | 54.96 | 10082.85 | 76.65 | 46.47 |
| W3 | 2017 | Agricultural | AGT | 3037.77 | 5.27 | 1303.00 | 2.26 | 0.36 | 934440 | 16.22 | 1.46 | 52.39 | 42.51 |
| W3 | 2017 | Bare Soil | BS | 446.58 | 0.78 | 273.00 | 0.47 | 0.13 | 151980 | 2.64 | 0.13 | 78.34 | 18.11 |
| **W3** | **2017** | **Oil palm crop** | **OPC** | **3009.24** | **5.22** | **179.00** | **0.31** | **2.70** | **291990** | **5.07** | **42.98** | **61.93** | **13.31** |
| W4 | 2001 | Rain forest | RF | 17965.08 | 31.19 | 1958.00 | 3.40 | 15.07 | 2619810 | 45.48 | **1348.68** | 62.41 | 49.25 |
| W4 | 2001 | Dry forest | DF | 377.64 | 0.66 | 393.00 | 0.68 | 0.07 | 163050 | 2.83 | 0.07 | 63.11 | 21.02 |
| W4 | 2001 | Shrubland | SHR | 2250.63 | 3.91 | 1338.00 | 2.32 | 0.67 | 624060 | 10.83 | 5.59 | 74.46 | 33.09 |
| W4 | 2001 | Hidrophytic vegetation | HV | 3644.19 | 6.33 | 1049.00 | 1.82 | 1.07 | 994890 | 17.27 | 9.29 | 50.64 | 41.42 |
| W4 | 2001 | Aquatic ecosystem | AQ | 5884.56 | 10.22 | 252.00 | 0.44 | 2.67 | 523620 | 9.09 | 64.40 | 61.20 | 17.28 |
| W4 | 2001 | Secondary vegetation | SV | 24366.51 | 42.30 | 1232.00 | 2.14 | 21.64 | 3029730 | 52.60 | 2861.03 | 69.65 | 49.25 |
| W4 | 2001 | Agricultural | AGT | 1928.43 | 3.35 | 695.00 | 1.21 | 0.36 | 528030 | 9.17 | 2.18 | 58.32 | 30.32 |
| W4 | 2001 | Bare soil | BS | 1182.96 | 2.05 | 342.00 | 0.59 | 0.60 | 254310 | 4.42 | 2.50 | 68.75 | 18.60 |
| W4 | 2017 | Rain forest | RF | 15063.75 | 26.15 | 1200.00 | 2.08 | 10.18 | 1829760 | 31.77 | **643.16** | 74.03 | 37.64 |
| W4 | 2017 | Shrubland | SHR | 2756.25 | 4.79 | 1539.00 | 2.67 | 0.37 | 896970 | 15.57 | 2.28 | 93.47 | 42.96 |
| W4 | 2017 | Hidrophytic vegetation | HV | 3071.25 | 5.33 | 1454.00 | 2.52 | 0.82 | 922410 | 16.01 | 4.77 | 69.88 | 41.79 |
| W4 | 2017 | Secondary vegetation | SV | 21769.83 | 37.79 | 982.00 | 1.70 | 13.86 | 2528730 | 43.90 | 1278.75 | 84.37 | 43.53 |
| W4 | 2017 | Agricultural | AGT | 4637.34 | 8.05 | 1043.00 | 1.81 | 1.18 | 948120 | 16.46 | 18.76 | 67.26 | 35.23 |
| W4 | 2017 | Bare soil | BS | 1416.42 | 2.46 | 238.00 | 0.41 | 0.42 | 225240 | 3.91 | 2.60 | 84.05 | 15.13 |
| **W4** | **2017** | **Oil palm crop** | **OPC** | **8885.16** | **15.43** | **217.00** | **0.38** | **2.78** | **682170** | **11.84** | **80.09** | **76.47** | **18.35** |
| W5 | 2001 | Rain forest | RF | 10669.05 | 18.52 | 1132.00 | 1.97 | 2.82 | 1642440 | 28.51 | **78.55** | 50.47 | 40.45 |
| W5 | 2001 | Dry forest | DF | 479.25 | 0.83 | 286.00 | 0.50 | 0.05 | 165630 | 2.88 | 0.06 | 41.75 | 19.09 |
| W5 | 2001 | Shrubland | SHR | 11214.18 | 19.47 | 2066.00 | 3.59 | 2.33 | 2199990 | 38.19 | 96.43 | 57.86 | 52.18 |
| W5 | 2001 | Hidrophytic vegetation | HV | 75.96 | 0.13 | 88.00 | 0.15 | 0.02 | 35130 | 0.61 | 0.00 | 46.70 | 9.98 |
| W5 | 2001 | Aquatic ecosystem | AQ | 516.42 | 0.90 | 69.00 | 0.12 | 0.35 | 113700 | 1.97 | 0.87 | 53.66 | 12.55 |
| W5 | 2001 | Secondary vegetation | SV | 28600.02 | 49.65 | 933.00 | 1.62 | 38.19 | 2904150 | 50.42 | 8421.41 | 62.62 | 43.61 |
| W5 | 2001 | Agricultural | AGT | 5675.04 | 9.85 | 1137.00 | 1.97 | 1.33 | 1252830 | 21.75 | 18.18 | 48.14 | 41.71 |
| W5 | 2001 | Bare soil | BS | 370.08 | 0.64 | 218.00 | 0.38 | 0.03 | 127530 | 2.21 | 0.04 | 55.99 | 16.52 |
| W5 | 2017 | Rain forest | RF | 20933.46 | 36.34 | 1001.00 | 1.74 | 5.44 | 2095020 | 36.37 | **575.65** | 60.08 | 36.89 |
| W5 | 2017 | Shrubland | SHR | 3175.65 | 5.51 | 692.00 | 1.20 | 1.53 | 540780 | 9.39 | 15.34 | 65.78 | 24.16 |
| W5 | 2017 | Hidrophytic vegetation | HV | 118.62 | 0.21 | 140.00 | 0.24 | 0.02 | 55530 | 0.96 | 0.01 | 66.71 | 12.74 |
| W5 | 2017 | Aquatic ecosystem | AQ | 488.43 | 0.85 | 44.00 | 0.08 | 0.44 | 98970 | 1.72 | 1.20 | 57.55 | 11.25 |
| W5 | 2017 | Secondary vegetation | SV | 16238.34 | 28.19 | 1543.00 | 2.68 | 3.03 | 2384250 | 41.39 | 106.86 | 51.60 | 47.27 |
| W5 | 2017 | Agricultural | AGT | 14786.64 | 25.67 | 1074.00 | 1.86 | 8.41 | 2157660 | 37.46 | 442.22 | 55.44 | 44.82 |
| W5 | 2017 | Bare soil | BS | 91.08 | 0.16 | 74.00 | 0.13 | 0.02 | 33930 | 0.59 | 0.01 | 76.09 | 8.88 |
| **W5** | **2017** | **Oil palm crop** | **OPC** | **1767.78** | **3.07** | **89.00** | **0.15** | **0.38** | **192420** | **3.34** | **2.62** | **51.14** | **11.41** |
| W6 | 2001 | Rain forest | RF | 16602.84 | 28.82 | 1864.00 | 3.24 | 5.66 | 2626200 | 45.59 | **335.07** | 67.42 | 51.52 |
| W6 | 2001 | Dry forest | DF | 2112.93 | 3.67 | 1145.00 | 1.99 | 0.28 | 703830 | 12.22 | 0.95 | 56.89 | 38.56 |
| W6 | 2001 | Shrubland | SHR | 466.56 | 0.81 | 336.00 | 0.58 | 0.10 | 152460 | 2.65 | 0.14 | 73.36 | 17.97 |
| W6 | 2001 | Hidrophytic vegetation | HV | 528.75 | 0.92 | 276.00 | 0.48 | 0.06 | 181260 | 3.15 | 0.11 | 57.02 | 19.82 |
| W6 | 2001 | Aquatic ecosystem | AQ | 1600.56 | 2.78 | 803.00 | 1.39 | 0.76 | 423180 | 7.35 | 6.72 | 81.92 | 26.56 |
| W6 | 2001 | Secondary vegetation | SV | 26582.49 | 46.15 | 1123.00 | 1.95 | 9.66 | 3348870 | 58.14 | 1138.51 | 75.13 | 51.96 |
| W6 | 2001 | Agricultural | AGT | 5370.57 | 9.32 | 2671.00 | 4.64 | 0.28 | 1934700 | 33.59 | 2.52 | 59.50 | 66.12 |
| W6 | 2001 | Bare soil | BS | 3575.34 | 6.21 | 2601.00 | 4.52 | 0.49 | 1417200 | 24.60 | 2.30 | 68.05 | 59.40 |
| **W6** | **2001** | **Oil palm crop** | **OPC** | **759.87** | **1.32** | **299.00** | **0.52** | **0.10** | **207000** | **3.59** | **0.20** | **72.93** | **18.80** |
| W6 | 2017 | Rain forest | RF | 13007.61 | 22.58 | 671.00 | 1.16 | 3.94 | 1304940 | 22.66 | **162.87** | 67.47 | 29.17 |
| W6 | 2017 | Dry forest | DF | 1062.18 | 1.84 | 399.00 | 0.69 | 0.21 | 286140 | 4.97 | 0.44 | 57.88 | 21.95 |
| W6 | 2017 | Shrubland | SHR | 1464.93 | 2.54 | 902.00 | 1.57 | 0.07 | 514230 | 8.93 | 0.21 | 71.48 | 33.87 |
| W6 | 2017 | Aquatic ecosystem | AQ | 1583.37 | 2.75 | 117.00 | 0.20 | 1.25 | 318570 | 5.53 | 12.56 | 84.34 | 20.10 |
| W6 | 2017 | Secondary vegetation | SV | 21732.57 | 37.73 | 822.00 | 1.43 | 10.25 | 2338080 | 40.59 | 870.59 | 77.13 | 40.27 |
| W6 | 2017 | Agricultural | AGT | 8379.45 | 14.55 | 780.00 | 1.35 | 5.69 | 1232190 | 21.39 | 193.84 | 47.97 | 33.95 |
| W6 | 2017 | Bare soil | BS | 557.37 | 0.97 | 195.00 | 0.34 | 0.09 | 144120 | 2.50 | 0.13 | 57.40 | 15.30 |
| **W6** | **2017** | **Oil palm crop** | **OPC** | **9812.52** | **17.04** | **210.00** | **0.36** | **13.32** | **373890** | **6.49** | **1030.34** | **62.17** | **9.67** |
| W7 | 2001 | Rain forest | RF | 24769.89 | 43.00 | 1369.00 | 2.38 | 18.41 | 3342660 | 58.03 | **2484.60** | 56.78 | 53.99 |
| W7 | 2001 | Dry forest | DF | 3910.05 | 6.79 | 2720.00 | 4.72 | 0.08 | 1484100 | 25.77 | 0.51 | 39.41 | 59.61 |
| W7 | 2001 | Shrubland | SHR | 15.48 | 0.03 | 30.00 | 0.05 | 0.01 | 8100 | 0.14 | 0.00 | 74.77 | 5.07 |
| W7 | 2001 | Aquatic ecosystem | AQ | 610.92 | 1.06 | 73.00 | 0.13 | 0.70 | 135600 | 2.35 | 3.28 | 74.15 | 13.78 |
| W7 | 2001 | Secondary vegetation | SV | 23728.05 | 41.19 | 1380.00 | 2.40 | 16.90 | 3408420 | 59.17 | 1807.15 | 59.43 | 55.70 |
| W7 | 2001 | Agricultural | AGT | 3371.13 | 5.85 | 1964.00 | 3.41 | 0.38 | 1168980 | 20.29 | 2.65 | 48.18 | 50.35 |
| W7 | 2001 | Bare soil | BS | 1190.16 | 2.07 | 1309.00 | 2.27 | 0.03 | 562830 | 9.77 | 0.06 | 64.11 | 40.91 |
| **W7** | **2001** | **Oil palm crop** | **OPC** | **4.32** | **0.01** | **9.00** | **0.02** | **0.00** | **2850** | **0.05** | **0.00** | **80.59** | **3.50** |
| W7 | 2017 | Rain forest | RF | 19027.35 | 33.03 | 1091.00 | 1.89 | 5.93 | 2227620 | 38.67 | **558.08** | 51.79 | 41.18 |
| W7 | 2017 | Dry forest | DF | 3060.63 | 5.31 | 1924.00 | 3.34 | 0.11 | 1090350 | 18.93 | 0.50 | 54.78 | 49.42 |
| W7 | 2017 | Shrubland | SHR | 840.78 | 1.46 | 908.00 | 1.58 | 0.03 | 374490 | 6.50 | 0.07 | 68.29 | 32.32 |
| W7 | 2017 | Aquatic ecosystem | AQ | 432.54 | 0.75 | 53.00 | 0.09 | 0.17 | 127500 | 2.21 | 0.59 | 78.99 | 15.37 |
| W7 | 2017 | Secondary vegetation | SV | 27029.79 | 46.93 | 873.00 | 1.52 | 17.56 | 3116820 | 54.11 | 1996.31 | 74.86 | 47.93 |
| W7 | 2017 | Agricultural | AGT | 2738.61 | 4.75 | 1056.00 | 1.83 | 0.40 | 736470 | 12.79 | 2.12 | 44.40 | 35.36 |
| W7 | 2017 | Bare soil | BS | 207.54 | 0.36 | 100.00 | 0.17 | 0.08 | 61620 | 1.07 | 0.06 | 66.29 | 10.64 |
| **W7** | **2017** | **Oil palm crop** | **OPC** | **4262.76** | **7.40** | **389.00** | **0.68** | **0.50** | **556590** | **9.66** | **6.44** | **54.02** | **21.35** |
| W8 | 2001 | Rain forest | RF | 25766.64 | 44.73 | 1511.00 | 2.62 | 24.33 | 2504100 | 43.47 | **3748.42** | 62.32 | 39.74 |
| W8 | 2001 | Dry forest | DF | 2453.04 | 4.26 | 1666.00 | 2.89 | 0.08 | 903360 | 15.68 | 0.34 | 47.62 | 45.67 |
| W8 | 2001 | Shrubland | SHR | 22.05 | 0.04 | 66.00 | 0.11 | 0.00 | 16440 | 0.29 | 0.00 | 76.91 | 8.59 |
| W8 | 2001 | Aquatic ecosystem | AQ | 977.85 | 1.70 | 215.00 | 0.37 | 0.92 | 208200 | 3.61 | 5.49 | 78.48 | 16.67 |
| W8 | 2001 | Secondary vegetation | SV | 21992.58 | 38.18 | 1123.00 | 1.95 | 19.46 | 3050490 | 52.96 | 2263.20 | 64.16 | 51.92 |
| W8 | 2001 | Agricultural | AGT | 4946.40 | 8.59 | 2194.00 | 3.81 | 0.48 | 1599960 | 27.78 | 3.70 | 52.02 | 57.19 |
| W8 | 2001 | Bare soil | BS | 1358.10 | 2.36 | 1452.00 | 2.52 | 0.08 | 630030 | 10.94 | 0.12 | 68.89 | 42.79 |
| **W8** | **2001** | **Oil palm crop** | **OPC** | **83.34** | **0.14** | **67.00** | **0.12** | **0.02** | **31320** | **0.54** | **0.01** | **84.62** | **8.56** |
| W8 | 2017 | Rain forest | RF | 10320.39 | 17.92 | 778.00 | 1.35 | 9.06 | 1084200 | 18.82 | **485.29** | 56.29 | 27.12 |
| W8 | 2017 | Dry forest | DF | 1075.23 | 1.87 | 579.00 | 1.01 | 0.11 | 348510 | 6.05 | 0.25 | 59.59 | 26.73 |
| W8 | 2017 | Shrubland | SHR | 1459.62 | 2.53 | 1023.00 | 1.78 | 0.07 | 518040 | 8.99 | 0.20 | 69.57 | 34.32 |
| W8 | 2017 | Aquatic ecosystem | AQ | 905.58 | 1.57 | 77.00 | 0.13 | 1.11 | 206250 | 3.58 | 7.22 | 77.56 | 17.17 |
| W8 | 2017 | Secondary vegetation | SV | 26777.34 | 46.49 | 687.00 | 1.19 | 20.27 | 2553480 | 44.33 | 2827.25 | 77.99 | 39.73 |
| W8 | 2017 | Agricultural | AGT | 7743.24 | 13.44 | 947.00 | 1.64 | 0.75 | 1342920 | 23.31 | 13.57 | 43.09 | 38.60 |
| W8 | 2017 | Bare soil | BS | 274.05 | 0.48 | 144.00 | 0.25 | 0.02 | 91920 | 1.60 | 0.02 | 72.10 | 13.81 |
| **W8** | **2017** | **Oil palm crop** | **OPC** | **9044.55** | **15.70** | **202.00** | **0.35** | **10.04** | **377760** | **6.56** | **616.05** | **55.45** | **9.99** |
| W9 | 2001 | Rain forest | RF | 27812.52 | 48.29 | 1390.00 | 2.41 | 24.31 | 3074340 | 53.37 | **4489.49** | 64.43 | 46.67 |
| W9 | 2001 | Dry forest | DF | 5152.95 | 8.95 | 1918.00 | 3.33 | 0.26 | 1430430 | 24.83 | 2.35 | 43.46 | 50.11 |
| W9 | 2001 | Shrubland | SHR | 20.79 | 0.04 | 40.00 | 0.07 | 0.00 | 12720 | 0.22 | 0.00 | 67.90 | 6.84 |
| W9 | 2001 | Aquatic ecosystem | AQ | 239.94 | 0.42 | 230.00 | 0.40 | 0.09 | 126360 | 2.19 | 0.09 | 85.40 | 20.31 |
| W9 | 2001 | Secondary vegetation | SV | 19748.07 | 34.28 | 1241.00 | 2.15 | 11.54 | 2848800 | 49.46 | 878.24 | 63.22 | 51.39 |
| W9 | 2001 | Agricultural | AGT | 2534.40 | 4.40 | 1955.00 | 3.39 | 0.16 | 1033590 | 17.94 | 0.55 | 53.91 | 51.50 |
| W9 | 2001 | Bare soil | BS | 1787.13 | 3.10 | 1314.00 | 2.28 | 0.17 | 665640 | 11.56 | 0.49 | 67.16 | 39.44 |
| **W9** | **2001** | **Oil palm crop** | **OPC** | **304.20** | **0.53** | **144.00** | **0.25** | **0.06** | **92340** | **1.60** | **0.05** | **73.87** | **13.18** |
| W9 | 2017 | Rain forest | RF | 6182.55 | 10.73 | 760.00 | 1.32 | 0.56 | 989430 | 17.18 | **8.24** | 55.23 | 31.77 |
| W9 | 2017 | Dry forest | DF | 46.71 | 0.08 | 35.00 | 0.06 | 0.02 | 18630 | 0.32 | 0.00 | 60.62 | 6.83 |
| W9 | 2017 | Shrubland | SHR | 1595.16 | 2.77 | 652.00 | 1.13 | 0.29 | 432690 | 7.51 | 0.98 | 69.66 | 27.18 |
| W9 | 2017 | Aquatic ecosystem | AQ | 332.28 | 0.58 | 82.00 | 0.14 | 0.11 | 115320 | 2.00 | 0.18 | 80.28 | 15.82 |
| W9 | 2017 | Secondary vegetation | SV | 17412.12 | 30.23 | 503.00 | 0.87 | 6.89 | 1546980 | 26.86 | 653.72 | 74.24 | 30.28 |
| W9 | 2017 | Agricultural | AGT | 1767.42 | 3.07 | 397.00 | 0.69 | 0.44 | 378120 | 6.56 | 2.21 | 42.20 | 22.67 |
| W9 | 2017 | Bare soil | BS | 235.71 | 0.41 | 72.00 | 0.13 | 0.09 | 57270 | 0.99 | 0.08 | 71.54 | 9.30 |
| **W9** | **2017** | **Oil palm crop** | **OPC** | **30028.05** | **52.13** | **307.00** | **0.53** | **48.35** | **711120** | **12.35** | **13473.45** | **48.34** | **10.62** |
| W10 | 2001 | Rain forest | RF | 16714.08 | 29.02 | 2253.00 | 3.91 | 3.62 | 3020820 | 52.44 | **211.14** | 57.82 | 59.21 |
| W10 | 2001 | Dry forest | DF | 4353.03 | 7.56 | 3302.00 | 5.73 | 0.22 | 1860180 | 32.29 | 1.31 | 51.30 | 70.93 |
| W10 | 2001 | Secondary vegetation | SV | 27601.65 | 47.92 | 1376.00 | 2.39 | 28.70 | 3812160 | 66.18 | 5062.98 | 67.60 | 57.87 |
| W10 | 2001 | Agricultural | AGT | 5376.33 | 9.33 | 1711.00 | 2.97 | 3.50 | 1459710 | 25.34 | 75.42 | 59.47 | 49.87 |
| W10 | 2001 | Shrubland | SHR | 120.51 | 0.21 | 247.00 | 0.43 | 0.01 | 74520 | 1.29 | 0.00 | 62.99 | 16.80 |
| W10 | 2001 | Hidrophytic vegetation | HV | 655.83 | 1.14 | 913.00 | 1.59 | 0.21 | 319440 | 5.55 | 0.38 | 50.43 | 31.19 |
| W10 | 2001 | Aquatic ecosystem | AQ | 54.27 | 0.09 | 107.00 | 0.19 | 0.01 | 41610 | 0.72 | 0.00 | 65.56 | 13.94 |
| W10 | 2001 | Bare soil | BS | 2723.04 | 4.73 | 1844.00 | 3.20 | 0.45 | 896460 | 15.56 | 2.54 | 63.45 | 43.05 |
| **W10** | **2001** | **Oil palm crop** | **OPC** | **1.26** | **0.00** | **4.00** | **0.01** | **0.00** | **1140** | **0.02** | **0.00** | **74.24** | **2.38** |
| W10 | 2017 | Rain forest | RF | 11782.17 | 20.46 | 1653.00 | 2.87 | 2.99 | 1986930 | 34.50 | **114.45** | 58.46 | 46.50 |
| W10 | 2017 | Dry forest | DF | 5049.45 | 8.77 | 1874.00 | 3.25 | 0.38 | 1413180 | 24.53 | 3.47 | 54.27 | 50.06 |
| W10 | 2017 | Shrubland | SHR | 1517.04 | 2.63 | 1589.00 | 2.76 | 0.07 | 713910 | 12.39 | 0.13 | 62.10 | 45.86 |
| W10 | 2017 | Aquatic ecosystem | AQ | 89.19 | 0.15 | 151.00 | 0.26 | 0.03 | 58350 | 1.01 | 0.01 | 84.52 | 15.52 |
| W10 | 2017 | Secondary vegetation | SV | 29331.45 | 50.92 | 806.00 | 1.40 | 31.10 | 2968230 | 51.53 | 5777.16 | 79.38 | 43.85 |
| W10 | 2017 | Agricultural | AGT | 3271.05 | 5.68 | 973.00 | 1.69 | 1.34 | 840780 | 14.60 | 11.85 | 48.71 | 36.85 |
| W10 | 2017 | Bare soil | BS | 299.88 | 0.52 | 252.00 | 0.44 | 0.05 | 114750 | 1.99 | 0.04 | 77.87 | 16.53 |
| **W10** | **2017** | **Oil palm crop** | **OPC** | **6259.77** | **10.87** | **220.00** | **0.38** | **2.14** | **384810** | **6.68** | **69.47** | **61.88** | **12.48** |
