# Supplementary material for: Tropical wetlands and land use changes: The case of oil palm in neotropical riverine floodplains
Source: PLoS One. 2022 May 12;17(5):e0266677. doi: 10.1371/journal.pone.0266677 (PMC9098095; doi:10.1371/journal.pone.0266677)

**S1 Figure 1. Land use/land cover change in subsection 1 (W1) Grijalva-Usumacinta freshwater ecoregion.**


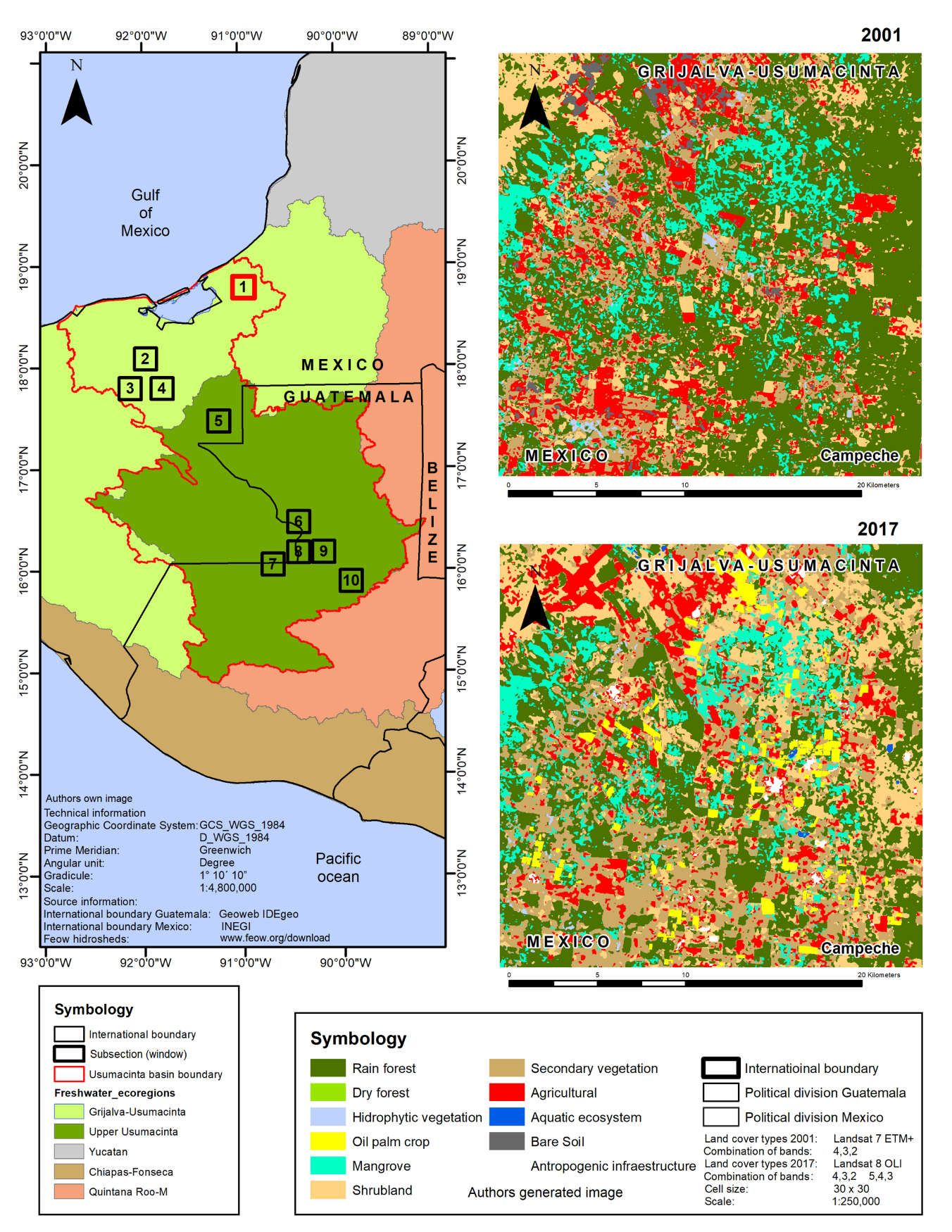


**S1 Figure 2. Land use/land cover change in subsection 2 (W2) Grijalva-Usumacinta freshwater ecoregion**


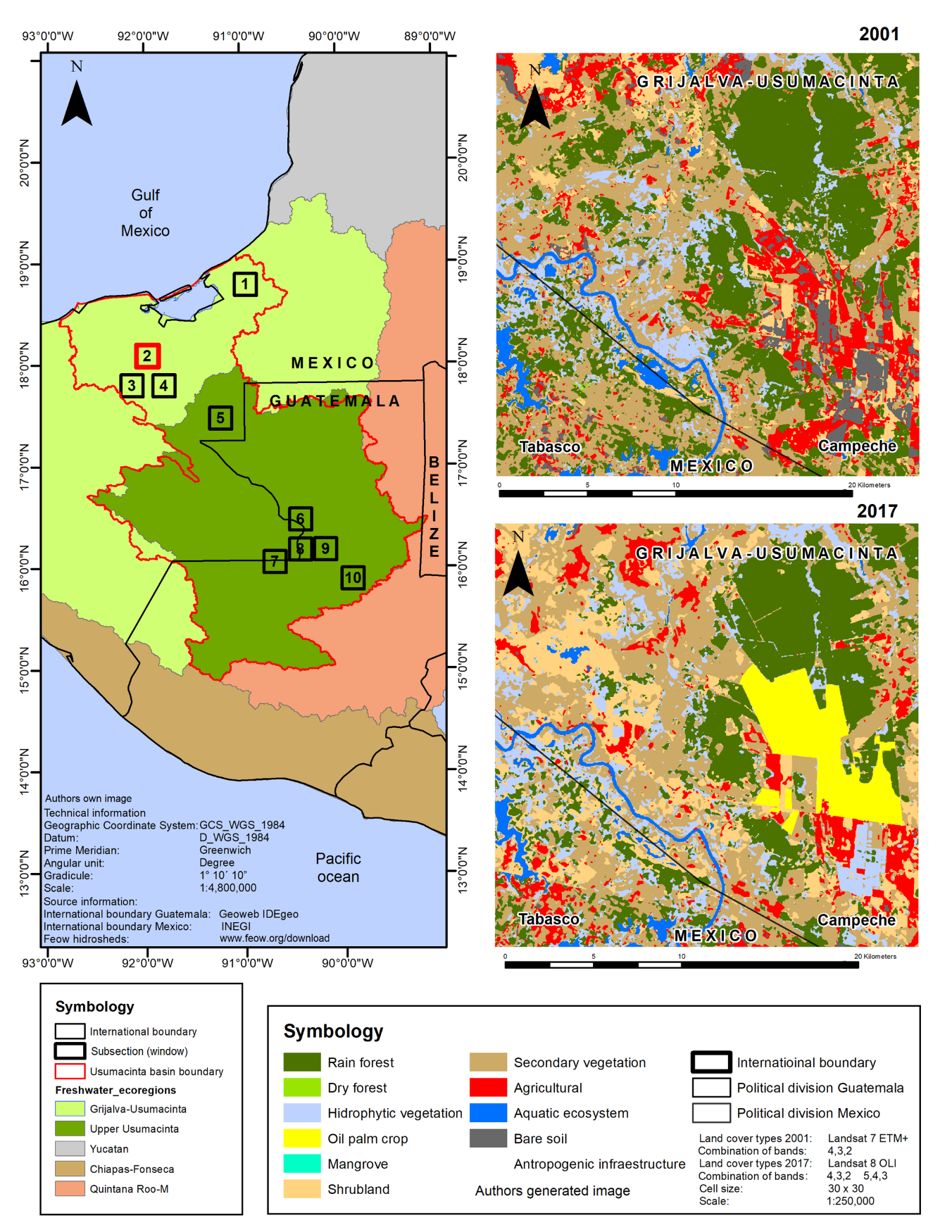


**S1 Figure 3. Land use/land cover change in subsection 3 (W3) Grijalva-Usumacinta freshwater ecoregion**


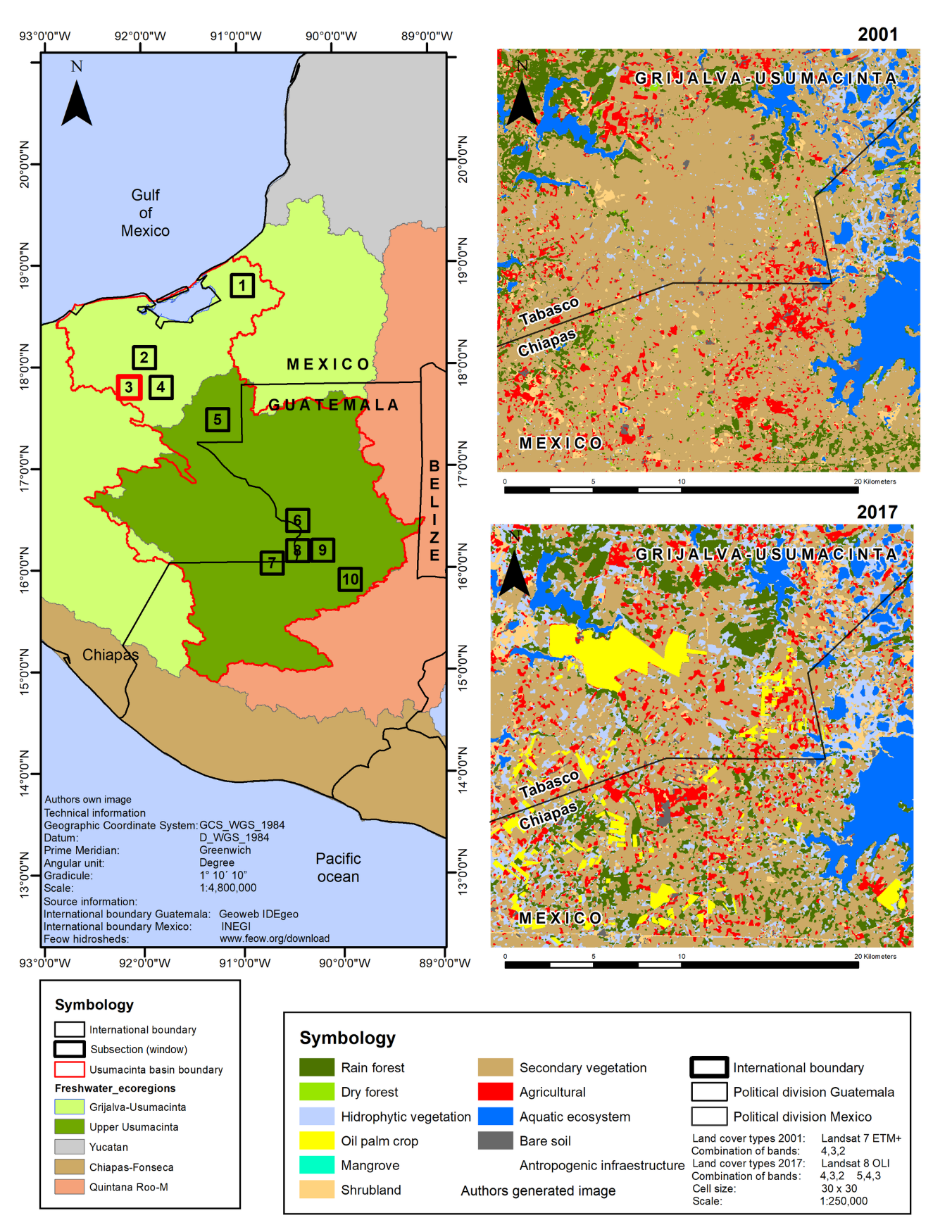


**S1 Figure 4. Land use/land cover change in subsection 4 (W4) Grijalva-Usumacinta freshwater ecoregion**


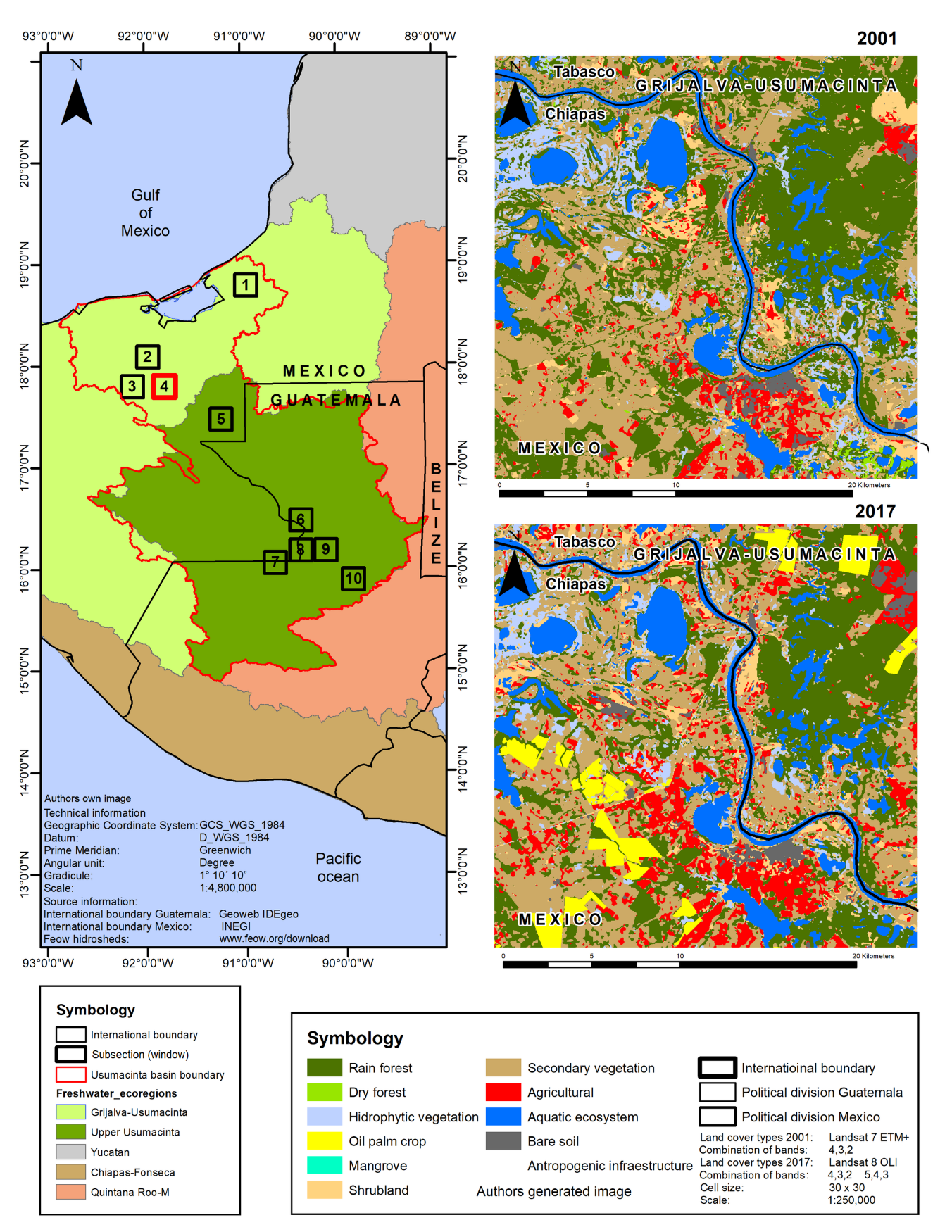


**S1 Figure 5. Land use/land cover change in subsection 5 (W5) Upper Usumacinta freshwater ecoregion**


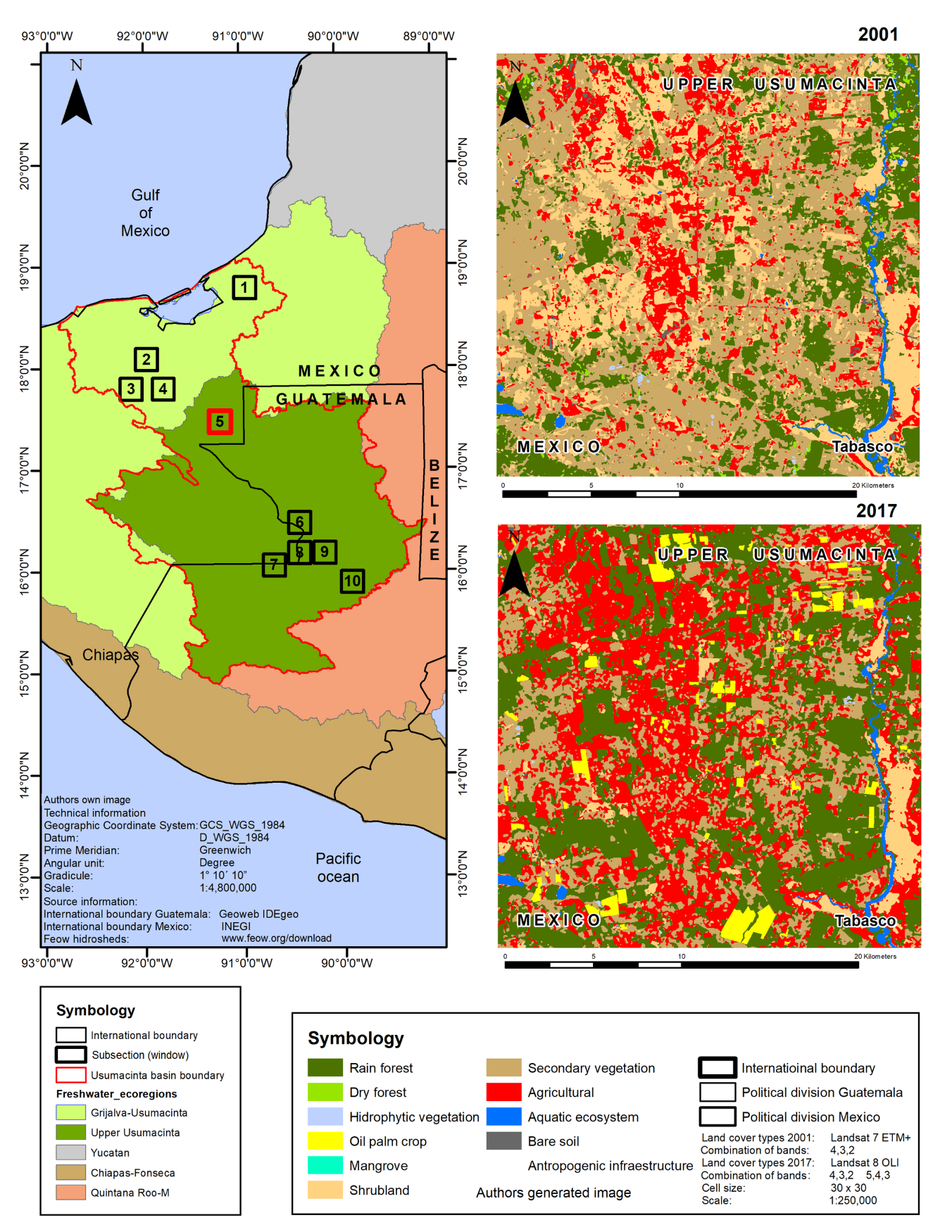


**S1 Figure 6. Land use/land cover change in subsection 6 (W6) Upper Usumacinta freshwater ecoregion**


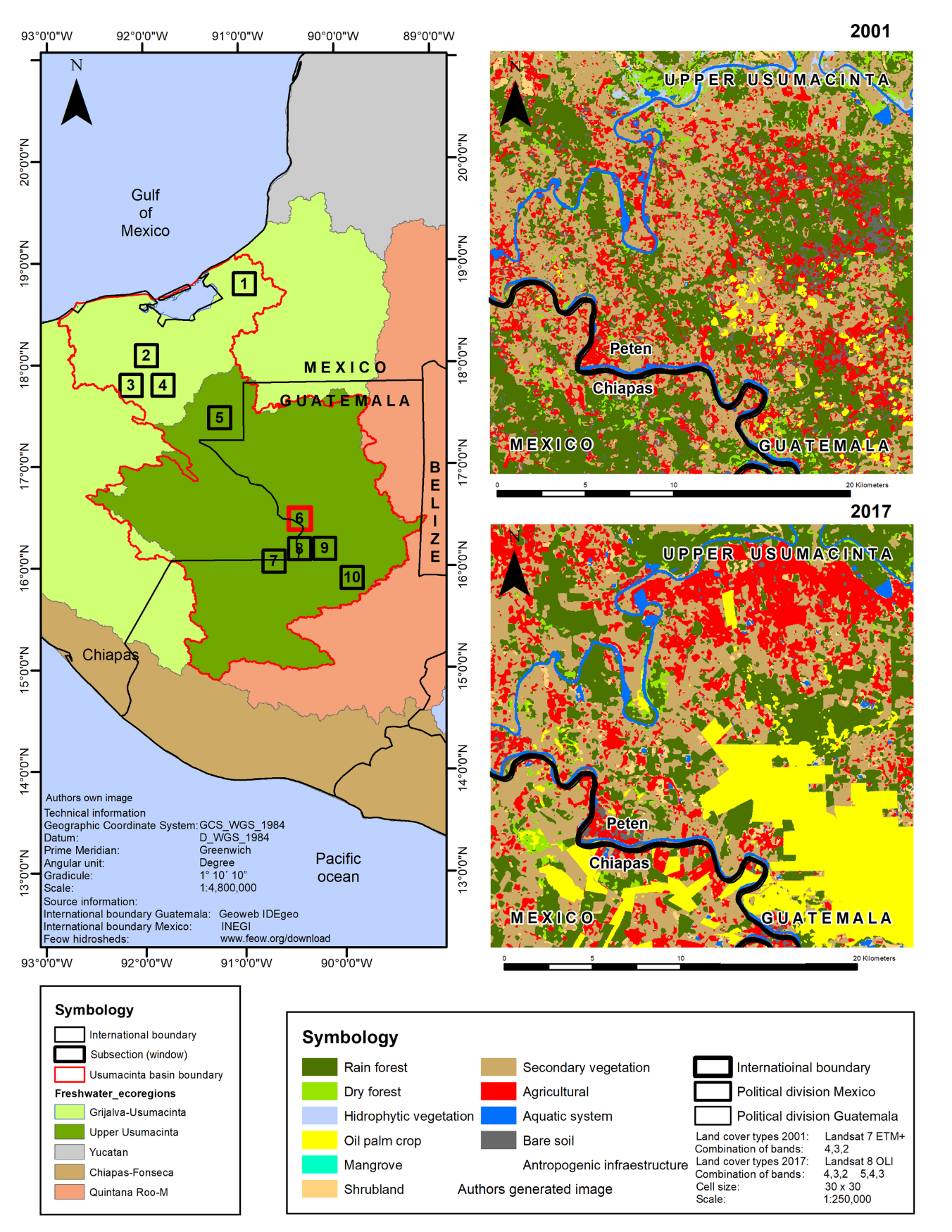


**S1 Figure 7. Land use/land cover change in subsection 7 (W7) Upper Usumacinta freshwater ecoregion**


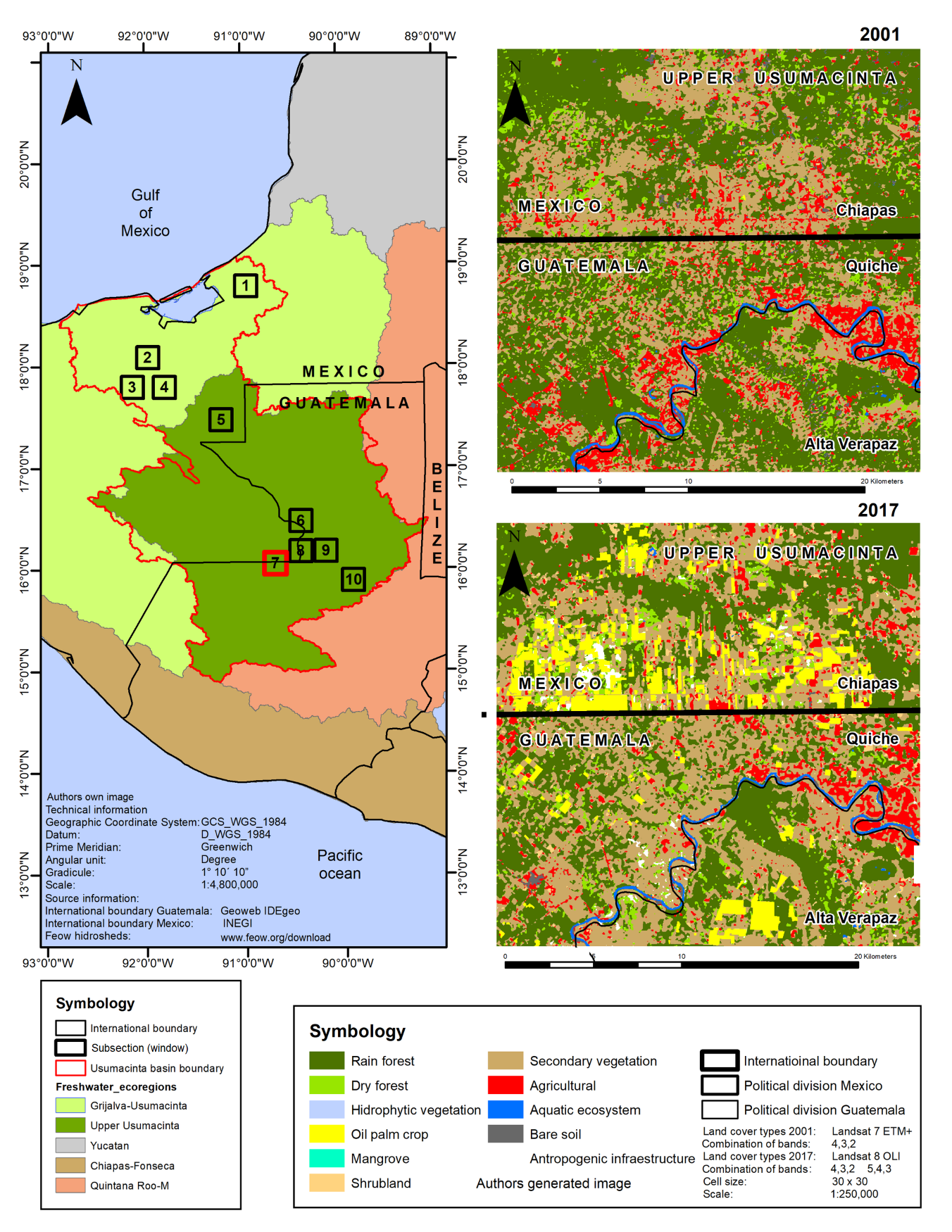


**S1 Figure 8. Land use/land cover change in subsection 8 (W8) Upper Usumacinta freshwater ecoregion**


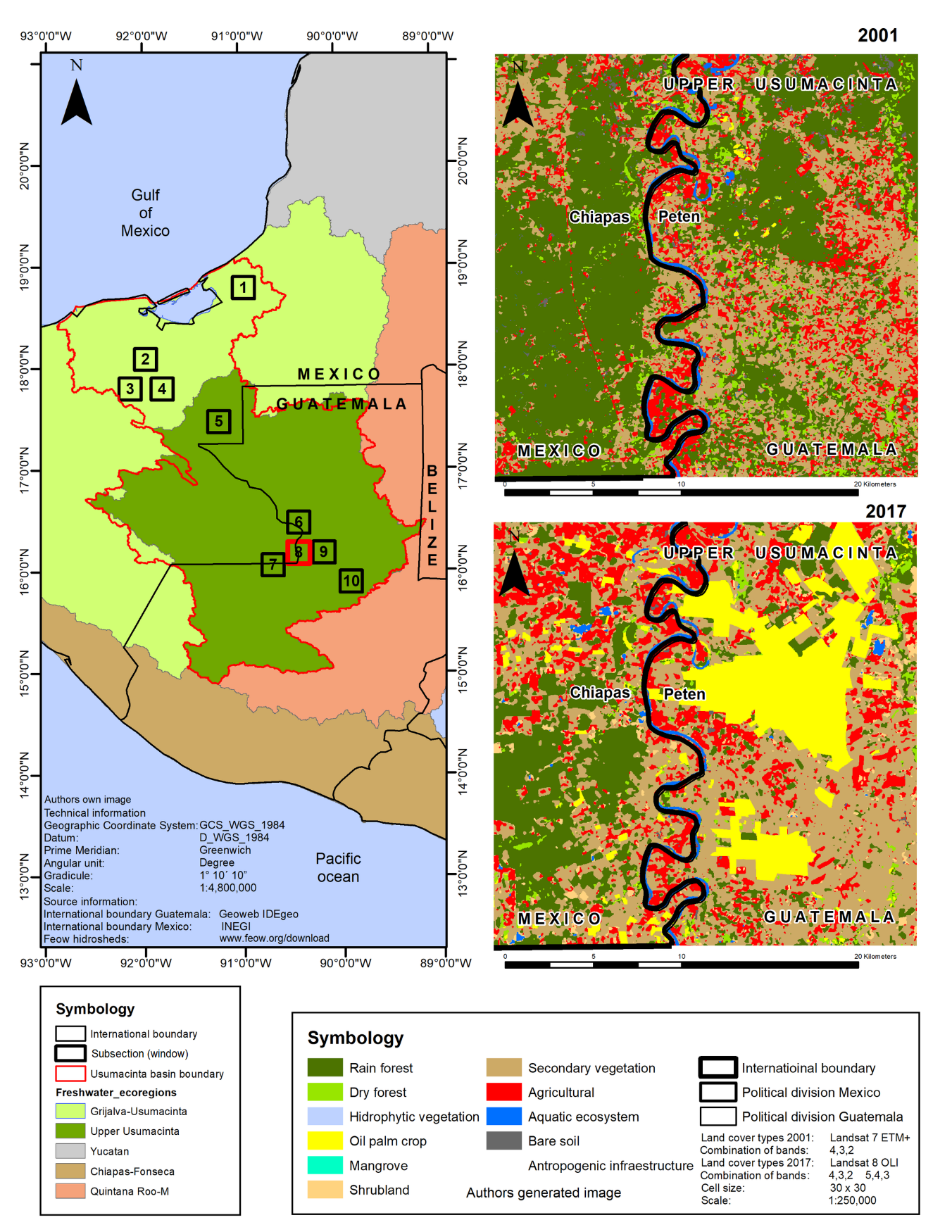


**S1 Figure 9. Land use/land cover change in subsection 9 (W9) Upper Usumacinta freshwater ecoregion**


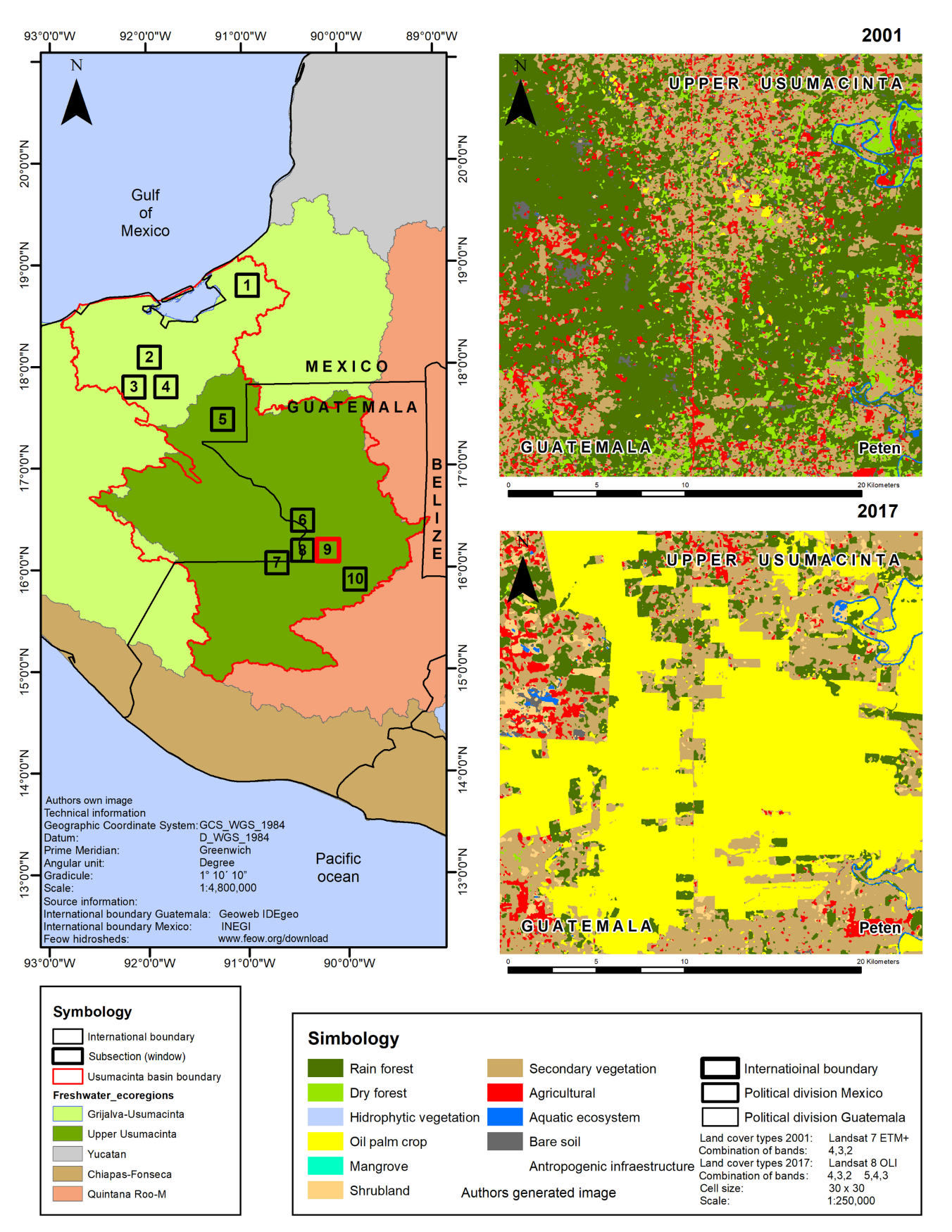


**S1 Figure 10. Land use/land cover change in subsection 10 (W10) Upper Usumacinta freshwater ecoregion**


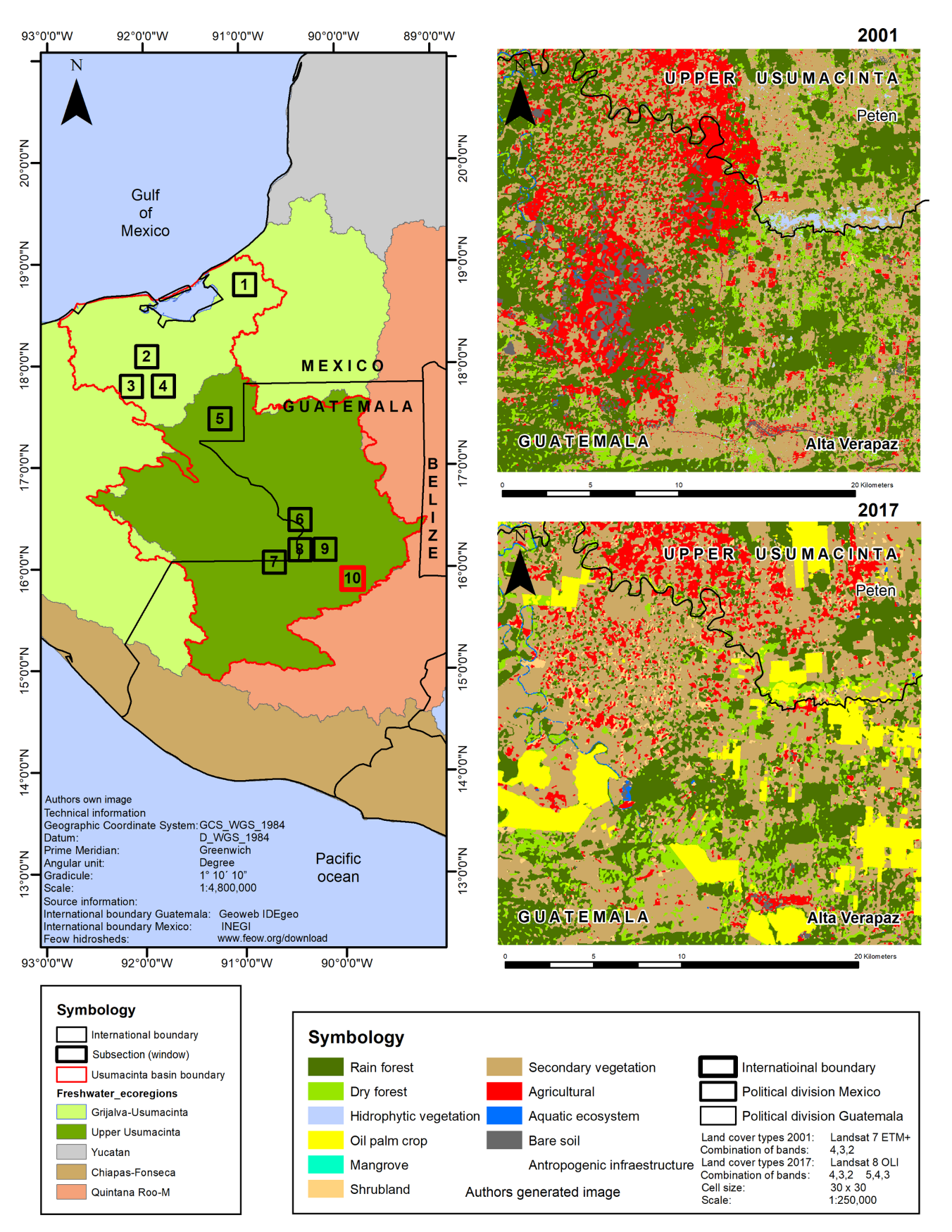

Supplement: S1 File — (DOCX) [file pone.0266677.s002.docx]
