# Supplementary material for: Tropical wetlands and land use changes: The case of oil palm in neotropical riverine floodplains
Source: PLoS One. 2022 May 12;17(5):e0266677. doi: 10.1371/journal.pone.0266677 (PMC9098095; doi:10.1371/journal.pone.0266677)
Supplement: Attachment — Submitted filename: Response to reviewers.docx [file pone.0266677.s003.docx]

RESPONSES TO REVIEWERS

**Manuscript number:** PONE-D-21-26813

**Title:** Tropical wetlands and land use changes: The case of oil palm in neotropical riverine floodplains..

**Authors:** Vera Camacho-Valdez, Rocío Rodiles-Hernández, Dario Navarrete-Gutiérrez, Emmanuel Valencia-Barrera

We are pleased to resubmit a revised version of our manuscript for publication. We appreciate the time and effort put forth by the editor and the constructive criticisms of the reviewers. We have addressed the issues indicated in the review process, and we believe that the revised version meets the journal’s publication requirements. We provide responses to the comments below.

# **JOURNAL REQUIREMENTS**

Dear Dr. Camacho-Valdez,

We've checked your submission and before we can proceed, we need you to address the following issues:

1. Thank you for providing information regarding the resources used to create your figures. Please include citations in your manuscript. For the image created using ArcGIS the creator ESRI requires either of the following statements: "Copyright © XXX. All rights reserved” or “Esri reserves the right to grant permission for any other use of the Image”.

Response:

**In the new version of the manuscript, we include in figure 2 the statement requested. The caption of figure is now as following:**

**Fig 2. Land use/land cover changes (2001-2017) generated with the GIS software ESRI ArcGIS 10.5. Copyright © 1995–2022 Esri. All rights reserved. Published in the United States of America. (Data source: Landsat 7 ETM+ and Landsat-8 OLI image courtesy of the U.S. Geological Survey).**

Dear Dr. Camacho-Valdez,

We've checked your submission and before we can proceed, we need you to address the following issues:

1. Thank you for providing additional information regarding your figures.
At this time, please kindly clarify the following regarding Figures 1, 2, and 4:

a) Where did the authors obtain the [maps, satellite images, basemaps, shapefiles, map data, etc.] in Figures 1, 2, and 4 ? Please also disclose what software was used to create these images.

Response:

For **figure 1** the autors obtain the information from:

**SHAPEFILES**

1. Usumacinta basin boundary polygon: shapefile of the polygon generated by the co-authors (DANG and EVB) through a SWAT process
2. Aquatic ecosystems Mexico: shapefile of drenage network downloaded free from INEGI (<https://antares.inegi.org.mx/analisis/red_hidro/siatl/>)
3. Aquatic ecosystems Guatemala: shapefile of drenage network downloaded free from SEGEPLAN (<http://ideg.segeplan.gob.gt/geoportal/>)
4. Oil palm crop: shapefile generated by the co-authors (DANG and EVB) from the digitalization of polygons in Google Earth
5. International boundary of Guatemala: Boundary shapefile downloaded free from SEGEPLAN (<http://ideg.segeplan.gob.gt/inicio/descargas.php>)
6. International boundary of Mexico: Boundary shapefile downloaded free from Marco Geoestadístico de los Estados Unidos Mexicanos Edición: 2021-INEGI (<https://www.inegi.org.mx/temas/mg/#Descargas>)

**RASTER**

7. DEM downloaded free from Digital Elevation - Shuttle Radar Topography Mission (SRTM) (<https://glovis.usgs.gov/app>)

**Figure 2.**

**SHAPEFILES**

5. The land use/land cover shape for **2001** was generated by the co-authors ( DANG and EVB) from the supervised classification of LANDSAT 7ETM+scenes dowloaded free from U.S. Geological Survey using glovis platform (<https://glovis.usgs.gov/>). USGS satellite imagery is public domain
6. The land use/land cover shape for **2017** was generated by the co-authors ( DANG and EVB) from the supervised classification of LANDSAT 8OLI scenes dowloaded free from U.S. Geological Survey using glovis platform (<https://glovis.usgs.gov/>). USGS satellite imagery is public domain.
7. International boundary of Guatemala: Boundary shapefile downloaded free from SEGEPLAN (<http://ideg.segeplan.gob.gt/inicio/descargas.php>)
8. International boundary of Mexico: Boundary shapefile downloaded free from Marco Geoestadístico de los Estados Unidos Mexicanos Edición: 2021-INEGI (<https://www.inegi.org.mx/temas/mg/#Descargas>)

**Figure 4**

**SHAPEFILES**

9. International boundary of Guatemala: Boundary shapefile downloaded free from SEGEPLAN (<http://ideg.segeplan.gob.gt/inicio/descargas.php>)
10. International boundary of Mexico: Boundary shapefile downloaded free from Marco Geoestadístico de los Estados Unidos Mexicanos Edición: 2021-INEGI (<https://www.inegi.org.mx/temas/mg/#Descargas>)
11. Usumacinta basin boundary polygon: shapefile of the polygon generated by the co-authors (DANG and EVB) through a SWAT process
12. Aquatic ecosystems Mexico: shapefile of drenage network downloaded free from INEGI (<https://antares.inegi.org.mx/analisis/red_hidro/siatl/>)
13. Aquatic ecosystems Guatemala: shapefile of drenage network downloaded free from SEGEPLAN (<http://ideg.segeplan.gob.gt/geoportal/>)
14. Oil palm crop: shapefile generated by the co-authors (DANG and EVB) from the digitalization of polygons in Google Earth
15. Freshwater ecoregions polygons: Freshwater Ecoregions of the World (FEOW) GIS shapefile obtained free from <https://www.feow.org/files/downloads/GIS_hs_snapped.zip>

In the new manuscript this information is already in the maps and figure captions. In fact, in figure 2 because we use Landsat imagery for LULC classification, we provided credit in the figure caption to the USGS as is recommended in their web page (<https://www.usgs.gov/centers/eros/data-citation?qt-science_support_page_related_con=0#qt-science_support_page_related_con>)

For the classification process we used IDRISI Selva software and for the maps production ArcGIS 10.5

b) Please state whether the [map/satellite images] have been previously copyrighted to your knowledge.

Response:

No, all the source of information that we use to generated our own maps are not copyrighted or come from públic domain web pages. In fact, all the maps generated for this manuscript are of their own authorship by two of the co-authors Dario Alejandro Navarrete Gutiérrez (DANG) and Emmanuel Valencia Barrera (EVB) from El Colegio de la Frontera Sur- Departamento Observación y Estudio de la Tierra, la Atmósfera y el Océano and are not copyrighted.

1. Please ensure that your manuscript meets PLOS ONE's style requirements, including those for file naming. The PLOS ONE style templates can be found at 
<https://journals.plos.org/plosone/s/file?id=wjVg/PLOSOne_formatting_sample_main_body.pdf> and 
<https://journals.plos.org/plosone/s/file?id=ba62/PLOSOne_formatting_sample_title_authors_affiliations.pdf>

2. In your Methods section, please **provide additional location information about your study area**, including geographic coordinates for the data set if available.

**Response**

**In the new version of the manuscript, we include the geographic coordinates as well as additional important information both in the study area map and in the text (lines 126-131).**

3. Thank you for stating the following financial disclosure:

**"The funders had no role in study design, data collection and analysis, decision to publish, or preparation of the manuscript."**

## QUERIES

a) Please clarify the **sources of funding** (financial or material support) for your study. List the grants or organizations that supported your study, including funding received from your institution.

**Response**

**This research was supported by the project “Conectividad y Diversidad Funcional de la Cuenca del rio Usumacinta” (FID-ECOSUR-784-1004).**

b) State what role the funders took in the study. If the funders had no role in your study, please state: “The funders had no role in study design, data collection and analysis, decision to publish, or preparation of the manuscript.”

**Response**

**“The funders had no role in study design, data collection and analysis, decision to publish, or preparation of the manuscript.”**

c) If any authors received a salary from any of your funders, please state which authors and which funders.

**Response**

**“The authors received no specific funding for this work.”**

d) If you did not receive any funding for this study, please state: **“The authors received no specific funding for this work.”**

**Response**

**“The authors received no specific funding for this work.”**

Please include your **amended statements** within your cover letter; we will change the online submission form on your behalf.

**Response**

**We include the previous statements in our submitted cover letter.**

4. We note that **Figures 1, 2 and 4 in your submission contain [map/satellite] images which may be copyrighted**. All PLOS content is published under the Creative Commons Attribution License (CC BY 4.0), which means that the manuscript, images, and Supporting Information files will be freely available online, and any third party is permitted to access, download, copy, distribute, and use these materials in any way, even commercially, with proper attribution. For these reasons, we cannot publish previously copyrighted maps or satellite images created using proprietary data, such as Google software (Google Maps, Street View, and Earth). For more information, see our copyright guidelines: <http://journals.plos.org/plosone/s/licenses-and-copyright>.

We require you to either (1) present written permission from the copyright holder to publish these figures specifically under the CC BY 4.0 license, or (2) remove the figures from your submission:
 
a. You may seek permission from the original copyright holder of Figures 1,2 and 4 to publish the content specifically under the CC BY 4.0 license.  
 
We recommend that you contact the original copyright holder with the Content Permission Form (<http://journals.plos.org/plosone/s/file?id=7c09/content-permission-form.pdf>) and the following text:
“I request permission for the open-access journal PLOS ONE to publish XXX under the Creative Commons Attribution License (CCAL) CC BY 4.0 (<http://creativecommons.org/licenses/by/4.0/>). Please be aware that this license allows unrestricted use and distribution, even commercially, by third parties. Please reply and provide explicit written permission to publish XXX under a CC BY license and complete the attached form.”
 
Please upload the completed Content Permission Form or other proof of granted permissions as an "Other" file with your submission.

In the figure caption of the copyrighted figure, please include the following text: “Reprinted from [ref] under a CC BY license, with permission from [name of publisher], original copyright [original copyright year].”

b. If you are unable to obtain permission from the original copyright holder to publish these figures under the CC BY 4.0 license or if the copyright holder’s requirements are incompatible with the CC BY 4.0 license, please either i) remove the figure or ii) supply a replacement figure that complies with the CC BY 4.0 license. Please check copyright information on all replacement figures and update the figure caption with source information. If applicable, please specify in the figure caption text when a figure is similar but not identical to the original image and is therefore for illustrative purposes only.

The following resources for replacing copyrighted map figures may be helpful:
 
USGS National Map Viewer (public domain): <http://viewer.nationalmap.gov/viewer/>

The Gateway to Astronaut Photography of Earth (public domain): <http://eol.jsc.nasa.gov/sseop/clickmap/>

Maps at the CIA (public domain): <https://www.cia.gov/library/publications/the-world-factbook/index.html> and <https://www.cia.gov/library/publications/cia-maps-publications/index.html>

NASA Earth Observatory (public domain): <http://earthobservatory.nasa.gov/>

Landsat: <http://landsat.visibleearth.nasa.gov/>

USGS EROS (Earth Resources Observatory and Science (EROS) Center) (public domain): [http://eros.usgs.gov/#](http://eros.usgs.gov/)

Natural Earth (public domain): <http://www.naturalearthdata.com/>

**Response**

**We provide new information in figures 1, 2, and 4 in order to comply with the CC BY 4.0 license. In the new version of the manuscript, we update the figure captions with the source information. With the information downloaded for free from different sources the authors generated their own images included in the text.**

**If we misunderstood the suggestion, and the editor did not mean this, we would be very grateful if she/he could provide further explanation.**

# **REVIEWER 1**

## GENERAL COMMENTS

This article presents valuable new information on the growing extent of oil palm plantations in a region of Latin America that has high ecological value. Such information will assist land managers and decision-makers in assessing the impacts of oil palm plantations, and to balance the trade-offs between economic development and ecosystem conservation. This article is timely given that the majority of the research focused on the impacts of palm oil plantations has been conducted in Asia, and yet as you correctly point out, such plantations have become established in other parts of the world and such trends are predicted to continue. The data reported are illustrated by high quality maps. I have made some suggestions for improvements in my detailed comments (below).

## SPECIFIC COMMENTS

Lines 15-16: To improve the language, I recommend amending "due to the global food demand and biofuels" to "due to the global demand for food and biofuels".

Line 16: The word "expenses" should be amended to "expense".

Line 17: The word "a" can be deleted.

Line 18: Amend "nearby" to "near to".

Line 20: Add the word "the" before "current".

Line 21: Add the word "a" before the phrase "case-study".

**Response**

**We appreciate your review and comments. We took these suggestions into account and the changes were made in the main text accordingly.**

Line 25: What are "intervened lands" in this context? Those previously impacted by people?

**Response**

**Yes, we referred to those land use/land cover that has been disturbed naturally or unnaturally, such as grazing, tree felling, frequent fires as well as land modified for agriculture.**

Line 26: It is not clear what the phrase "aquatic ecosystem cover" means in this context. Do you mean the spatial extent of aquatic ecosystems? It is clearly an important concept for your study, and so it would be helpful to the reader to define it.

**Response**

**Yes, “aquatic ecosystem cover” refers to the spatial extent of aquatic ecosystems that includes rivers, lagoons and channels. In the abstract we change the word “cover” by “class” for a better clarity. We include also in parenthesis the ecosystems included in this class.**

Lines 26-27: "Although aquatic ecosystem cover decreased in surface during the study period". The abstract would be more informative if you would state by how large this decrease was. What % of the initial area was lost? The information from Table 3 could be used here.

**Response**

**Following the suggestions of the reviewer we have rewritten the abstract for better clarity, highlighting the percentage of change of aquatic ecosystem class.**

Line 43: Does "production" here refer to annual production? Please clarify.

**Response**

**Yes, we referred to the annual production of palm oil in Indonesia. We include at the beginning of the paragraph the word “annual” for a better understanding of the context.**

Line 43: Amend "increase" to "increased".

Line 50: Amend "has" to "have".

Line 134: I recommend that here you clarify that LULC refers to "land use land cover", as readers that look at the figures first may have missed this definition in the text.

Line 344: Amend "decrease" to "decreasing".

Line 350: Change "much" to "far".

Line 351: The word "a" should be changed to "the", and the word "are" should be amended to "is".

**Response**

**We appreciate your review and comments. We accept these suggestions and the text was modified accordingly.**

Lines 362-363: "The effects on sediment and water quality can extend over comparatively large distances". It would be useful to explain more detail what effects increased sediment inputs might have on water quality and aquatic ecosystems. In particular, the point should be made that high sediment inputs to aquatic ecosystems can increase turbidity, which negatively affects aquatic plants, which are often the key primary producers in aquatic ecosystems (O’Hare, M.T., et al. 2018. Plants in aquatic ecosystems: current trends and future directions. Hydrobiologia, 812, 1-11.). Sediment inputs can therefore have substantial impacts on the structure and functioning of aquatic ecosystems, which may ultimately affect their provision of ecosystem services to local communities (Mitsch, W.J., et al. 2015. Ecosystem services of wetlands. International Journal of Biodiversity Science, Ecosystem Services & Management, 11, 1-4.).

**Response**

**In the new version of the manuscript, we included a paragraph (lines 366-372) where the information regarding the effect of sediment on aquatic systems is complemented and supported with the literature recommended by the reviewer as well as other studies.**

Line 378: This is an important point, because fish communities are known to be influenced by the forests within their catchment (Lo, M., et al. 2020. The influence of forests on freshwater fish in the tropics: A systematic review. BioScience, 70, 404-414.), and so the conversion of native rainforest to oil palm plantations will likely have impacts on the in-stream fish communities in this region.

**Response**

**We have included a paragraph (lines 389-392) which better describes the influence of forest on fish communities using this as it is an interesting example of the impact of deforestation on freshwater fishes.**

Lines 762-766: There is an error with the formatting of the references here. There are currently two reference #37s and two reference #38s.

**Response**

**Thank you very much, we removed the two references that were not cited in the text.**

# **REVIEWER 2**

## GENERAL COMMENTS

The expansion of oil palm plantations is a global issue. This study used Landsat 8 OLI images coupled with regular geostatistics to quantify the spatial distributions of oil palm area and the land-use changes of different landscapes (agricultural, oil palm crop, aquatic ecosystem, etc..) over 17-year plantation history (from 2001-2017). As a case study, the present work alerts the audiences to the serious problem in a biodiversity hotspot (Usumacinta River Basin), in face to the accelerated oil palm plantation processes.

**We thank the reviewer for the time spent to review this manuscript and for the comments.**

## MAJOR COMMENTS

(1) Although the present study has used several geostatistical tools to reveal the landscape patterns in the Usumacinta watershed, the effects of oil palm crops on floodplain ecosystems remained largely descriptive, without substantial data supporting them (e.g., lines of the evidence mostly derived from previous pieces of literature). Therefore, the objectives of this study should be better framed.

**Response**

**We very much agree with the reviewer. We have included a landscape analysis which better describes the effects of oil palm on floodplain ecosystems (Lines 236-273; 393-443; 451-454). Details about the specific changes can be seen in the following responses.**

(2) To well address the above issue, I suggest further quantifying the **fragmentation degree of landscape and/or landscape heterogeneity characteristics**, as well as other commonly-used geographical indices before and after large-scale oil palm plantation, based on the currently obtained data points. For instance, how “environmental integrity” is impaired by oil palm plantation, since the authors stated in multiple places the plantation could affect habitat complexity, environmental integrity, and connectivity, and highlighted the importance of structurally complex ecosystems of naturally-occurring tropical forest. By doing this, the authors can **provide robust evidence** to support the arguments about the negative impacts of oil palm plantations.

**Response**

***Methods***

**In the new version of the manuscript, we have included in the methods another section with the steps we follow for the “landscape analysis” suggest by the reviewer (Lines 236-273). We first defined ten subsections (windows) distributed throughout the study area to evaluate the differences between the composition and configuration of the landscape before and after the establishment of oil palm crop. In the main text are described the criteria used to define these subsections. We consider fragmentation, heterogeneity and diversity landscape metrics. Finally, we performed a Standardized Principal Component Analysis (PCA) to assess changes in the subsections previous defined, considering the metrics calculated.**

***Results***

**We improved the results section including the findings related with the fragmentation indices highlighting the effect of oil palm cultivation on the landscape heterogeneity (Lines 393-443). In this new version of the manuscript we include a table (table 4) with the landscape metrics obtained as well as a figure with the information related with the PCA. Supplementary material is also included in order to support and complement our main findings related with the landscape analysis (S1 figure and S1 table documents).**

***Discussion***

**We provide information in the discussion section that better support our landscape main findings (Lines 451-456).**

***Observation***

**It is important to mention that during the analysis of the landscape in the defined subsections, we found the presence of oil palm during 2001. We had not presented this finding in the previous submitted version. Although it does not represent a significant change, since there are few hectares of oil palm (2,987 ha), table 3 is updated in the new version of the manuscript. We also modified the description in the main text that corresponds with the updated table.**

(3) Some technical issues should be addressed before publication: a) lines 180-181, the cloud cover data for the images used should be provided; b) Landsat 8 satellite was only launched after 2013, how can obtain images using Landsat 8 for those in 2011-2012; c) line 182, more information about atmospheric and geometrical correction should be provided. For instance, what models have been adopted for atmospheric correction?

**Response**

a) **We have added information about the cloud data we used to select images (lines 186-187).**
b) **Thank you very much for this observation. In the previous manuscript we forgot to include the other type of image that we used for the 2001 classification. In the new version of the manuscript this information is included (lines 182-184)**
c) **We present further information in the text about the tools and models used to perform the atmospheric and geometrical correction (lines 191-203).**

(4) There is a **combined result and discussion in the Result section**. Please make a clear separation between your results and discussion, and be sure the discussion answers what the introduction asked.

**Response**

**We appreciate the reviewer comment. However, after reading the results, we consider that they are written quite specifically and concrete. Only in the first section referred with the ES identified and the potential effect of oil palm crops (lines 221-282) there is a broader description of the findings that we think is more suitable for the Results section rather than the Discussion.**

**In addition, in the new version of the manuscript, we include information in the discussion section that better support our main findings.**

**If we misunderstood the suggestion, and the reviewer did not mean this, we would be very grateful if she/he could provide further explanation.**

## MINOR COMMENTS

1. Line 44, oil palm production in 2010 and 2011 has been reported. What about the current conditions (e.g., in 2020 or the recent five years)?

**Response**

**We appreciate your review and comments. We include information in the new manuscript related with the current condition of oil palm production (2020). We support the data with references.**

2. Lines 48-49, “8.9 billion tons in 2050”, it means globally or in Indonesia? Please specify.

**Response**

**Yes, we referred to the global production of oil palm. We include in the text the word “global” for more clarity.**

3. Line Abstract & 21 & 110, add “a” before case-study site

**Response**

**We appreciate your review and comments. We accept these suggestions and the changes were made to the main text accordingly.**

4. Lines 114-118, the study aim should be framed more logically. For example, the vertical distribution of oil palm plantations, land-use conversion, as well as the spatial distribution of oil palm crops by freshwater ecoregion have been studied, but not been clearly stated here.

**Response**

**We appreciate your review and comments. We accept this suggestion and the text has been modified (Lines 115-121), stating more clearly the main questions related with the aim of the study.**

5. Line 128, remove the comma before [52].

6. Line 129, add a colon between “floodplains” and “a deltaic one”.

7. Lines 169-170, rephrase this sentence.

**Response**

**We accept these suggestions and the text was modified accordingly.**

8. Line 203, “Table 2” should not be ahead of “Table 1”.

**Response**

**Thank you very much for your comment. To avoid confusion with the order of the tables in the new version of the manuscript we removed the paragraph “A brief description of the land cover categories is given in Table 2”. This hopefully allows a better understanding of the text.**

9. Lines 251-252, your data points cannot support your statements “we found that the greatest environmental impact comes from pollution and habitat fragmentation due to deforestation”. Besides, a citation of this sentence means that you cited previous reports to support your viewpoints.

**Response**

**We agree with the reviewer that our data point cannot support our statement “we found that the greatest environmental impact comes from pollution and habitat fragmentation due to deforestation”. In fact, this section is a brief description of the "potential" impacts of oil palm on riverine floodplains and is based on few studies we found, but we believe it helps introduce the reader to the issue of oil palm and its effects.**

**We modified the text including only an overall description of the oil palm impacts supported with the reviewed studies (lines 253-255).**

10. Line 301, from Table 3, how can the increased oil palm areas be contributed to a net loss of 39% area of rainforest?

**Response**

**Thank you very much for this observation. Indeed, this data (39% net loss) cannot be observed in table 3 so we remove it from the text in this new version of the manuscript. In fact, this data is mentioned in the section where we describe the land cover that was converted to oil palm (line 322-326). The percentage converted to palm oil during the study period (2001-2017) is calculated for each cover type coverage and this can be observed in figure 5.**

11. Lines 401-403, perhaps the authors can give a simple calculation to account for this conclusion. What is the balance between the production of bioenergy in form of carbon and the loss of biomass carbon?

**Response**

**We provided information on this issue supported with the relevant literature (lines 414-423). If we misunderstood the suggestion, and the reviewer did not mean this, we would be very grateful if she/he could provide further explanation.**
